# Advances in systems immunology and cancer

**DOI:** 10.3389/fphys.2014.00249

**Published:** 2014-07-02

**Authors:** Kumar Selvarajoo

**Affiliations:** ^1^Systems Immunology, Institute for Advanced Biosciences, Keio UniversityTsuruoka, Japan; ^2^Systems Biology Program, Graduate School of Media and Governance, Keio UniversityFujisawa, Japan

**Keywords:** systems biology, high dimensional data, immunology, cancer, plasticity, computational biology, statistics, nonparametric

The last two decades have generated numerous studies that show the close link between immune response and cancer progression in the mammalian system. In parallel, we have also witnessed significant progress in systemic approaches, such as high-throughput, multi-dimensional and dynamical analyses, in tackling biological complexities. We took this opportunity to organize a research topic that encompasses the current advances in immunology and cancer. The intention is to emphasize the importance of holistic view, and how such outlook can help shape the future of biological research. In total, our topic consists of 10 articles: five reviews, three research, and two perspectives.

Owens and Naylor introduces the current understanding of cancer heterogeneity and stemness (Owens and Naylor, 2013). They surveyed a depth of recent literature that points to the presence of breast cancer stem cells (CSCs), which are responsible to mediate metastasis and are resistant to both radiation- and chemo-therapies. Although the classification of CSCs are currently based on the expression levels of cell surface markers CD44^+^CD24^−^ and enzyme Aldehyde dehydrogenase (ALDH) activity, they note that the heterogeneity of single cancer cells makes this classification a nontrivial process. Thus, they ask for more mechanistic approaches to elucidate the origins for CSCs, so that more targeted novel therapies can be developed.

In another survey of cancer mechanisms, Catalan et al. discuss the importance in understanding the connection between adipose tissue immunity and cancer (Catalán et al., 2013). They first quote numerous works that showed obesity-related chronic inflammation and, next, mention others that have demonstrated increased levels of immune cells and proinflammatory mediators in the expanded adipose tissue. Finally, they note specific obesity-associated adipokines that can promote tumor growth. Although the mechanistic links between obesity and cancer still remains unclear, more systemic analyses could reveal better hints in the future.

Sangdun Choi and colleagues present a detailed update on the different structures of the crucial innate immune pattern recognition receptors, namely the Toll-like receptors (TLRs) (Manavalan et al., 2011). There are 13 known mammalian TLRs to-date, however, details of TLR12 and 13 is vastly unclear. Here, the authors cover the details of TLR1-11, especially on their structures, to understand the interactions of TLRs with their ligands and activators. They also argue that 3-D molecular simulations can be useful to make predictions on unknown interactions between TLRs and other possible novel interacting partners.

Remaining on the same topic of TLRs, to investigate the differential roles of adaptor molecule MyD88 and MAP kinase activation in early and late immune response, which will influence the spatial movement of macrophages, Wenzel et al. developed an intelligent algorithm for automated image analysis (Wenzel et al., 2011). The novel approach is able to track cell spreading, after ligand stimulation, more accurately and with significant improvement in processing time, compared with manual techniques that are commonly adopted. Their main findings indicate that MyD88 is key for late spreading of macrophages while MAP kinase p38 is crucial for early spreading.

Apart from innate immunity, another important aspect of our immune response is the orchestration of the adaptive immunity. T cells are lymphocytes that are central in the adaptive responses. In order to perform its specialized task, T cells need to differentiate into different lineages for executing distinct responses. In the unstimulated naïve form, T cells exist mainly as two subtypes depending on their surface markers, CD4^+^, and CD8^+^ T cells. Ganusov and colleagues reviewed the differentiation lineages taken by CD4^+^ T cells on encountering MHC class II found on the surface of antigen-presenting cells such as macrophages or dendritic cells (Magombedze et al., 2013). Mainly, they emphasize on the functional plasticity of CD4^+^ T cells, and argue that understanding this will help treat diseases such as autoimmune diseases and allergic reactions where the elevated activity of differentiated T cells (e.g., T helper 17 or Th17 cells) may be reprogrammed to reach a different attractor state or back to its naïve form that will not be injurious to the host. They acknowledged that computational or mathematical models can be useful for predicting how one could convert a particular T cell subset into another.

A subsequent manuscript by Blair et al. reviews some of the most common mathematical and statistical approaches used for immune and cancer systems biology at different scales of biological modularization (Blair et al., 2012). Next, Hiroi and colleagues present a method to optimize the model parameters where experimental data are either sparse or noisy (Hiroi et al., 2014). They tested their method on well-established data on c-Myc and E2F transcriptional processes. The following article by Oyama and colleagues briefly discusses about recent high-throughput phosphoproteomics research (Kozuka-Hata et al., 2012). They describe the basic terminologies used and also highlight the importance of such methods for the development of large-scale signal transduction models for systemic interpretation of EGF signaling, TLR signaling or any other pathways of interest.

Fitting with the theme of adopting systemic approaches for understanding immune and cancer response is the paper by Campbell et al. (2011). Here, they have studied the distinct roles of CD8^+^ T cells to the pathogenesis of cancer. Using *in vivo* derived quantitative data of tumor promoting Tag-expressing mice cells encountering CD8^+^ T cells, they developed a computational model to investigate the interaction pathways. Remarkably, using a simple ordinary differential equation model, the responses of CD8^+^ T cells to different perturbations *in silico* were consistent with matched experiments. However, from the model, it became clear that the proliferation and decay rates of CD8^+^ T cells were strongly constrained and hence, Tag-expressing mice cells become tolerant to tumors. Knowing such information *a priori* will surely aid researchers to understand and possibly avoid poor targets for regulating cancer progression.

Finally, we conclude our collection with an interview with a prominent Japanese physicist, Kaneko (2011), who has switched his interest from pure theory to understanding complex living systems. In the article, he describes the reason behind his renewed interest, and the challenges facing theoreticians in biology. In summary, we believe the articles in “Advances in Systems Immunology and Cancer” research topic or e-book will bring continued interests for the development and utility of multidisciplinary approaches to tackle complex diseases.

## Conflict of interest statement

The author declares that the research was conducted in the absence of any commercial or financial relationships that could be construed as a potential conflict of interest.
